# Proanthocyanidins Alleviate T-2 Toxin-Induced Toxicity in Yak (*Bos grunniens*) Sertoli Cells by Alleviating Oxidative Stress and Modulating Mitochondrial Biogenesis

**DOI:** 10.3390/antiox15050547

**Published:** 2026-04-25

**Authors:** Huai Zhang, Dongju Liu, Linwen Ding, Fuchao Zhang, Jianmei Mao, Wanzhong He, Qilin Zhuoma, Honghong He, Wei Fu, Daoliang Lan, Shi Yin

**Affiliations:** 1 College of Animal and Veterinary Sciences, Southwest Minzu University, Chengdu 610041, China; 230951332003@stu.swun.edu.cn (H.Z.); 230905222002@stu.swun.edu.cn (D.L.); 250951332011@stu.swun.edu.cn (L.D.); 220952002019@stu.swun.edu.cn (F.Z.); honghong3h@swun.edu.cn (H.H.);; 2 Jiajiang County Agriculture and Rural Bureau, Leshan 614100, China; 3 Jingyan County Agriculture and Rural Bureau, Leshan 614100, China; 4 Diqing Tibetan Autonomous Prefecture Productivity Promotion Center, Diqing 674400, China

**Keywords:** T-2 toxin, proanthocyanidins, Sertoli cell, oxidative stress, mitochondrial

## Abstract

T-2 toxin, a mycotoxin produced by the genus *Fusarium*, is widely prevalent in agricultural products and livestock feed, posing substantial health risks to livestock and humans. This toxin induces oxidative stress in testicular Sertoli cells, disrupts testicular architecture, and compromises spermatogenesis. Despite its widespread presence in contaminated feeds, effective therapeutic strategies to counteract T-2 toxin-induced reproductive toxicity in Sertoli cells remain elusive. This study evaluated the protective efficacy and molecular mechanisms of proanthocyanidins (PCs), a phytochemical with antioxidant properties, against T-2 toxin-induced damage in yak (*Bos grunniens*) Sertoli cells. The findings revealed that T-2 toxin markedly reduced the viability of yak Sertoli cells and stimulated the production of reactive oxygen species (ROS). Treatment with 10 μg/mL PCs significantly enhanced cell viability, decreased apoptosis, and preserved cellular functions. Furthermore, PCs reduced ROS levels in yak Sertoli cells exposed to T-2 toxin and improved antioxidant capacity by upregulating the nuclear factor erythroid derived 2-like (NRF2)/heme oxygenase-1 (HO-1) signaling pathway. Additionally, PCs inhibited mitochondria-mediated apoptosis, diminished the occurrence of malformed mitochondria, and enhanced the sirtuin 1 (SIRT1)/peroxisome proliferator-activated receptor gamma coactivator 1 alpha (PGC-1α) signaling pathway associated with mitochondrial biogenesis in yak Sertoli cells exposed to T-2 toxin. This study provides novel insights into the prevention and treatment of T-2 toxin-induced reproductive damage in yaks and underscores the potential application of PCs in this context.

## 1. Introduction

Sertoli cells (SCs), the only somatic cells in direct contact with spermatogenic cells within the testis, are vital for supporting spermatogenesis through structural support, nutritional provision, and immune protection [1,2,3]. Tight junctions between adjacent Sertoli cells constitute the blood-testis barrier (BTB). This physical barrier isolates developing spermatogenic cells from the immune system and restricts the entry of bacteria and other pathogens. Consequently, it assists in the establishment and maintenance of a stable microenvironment necessary for successful spermatogenesis [4,5]. Additionally, SCs regulate spermatogenesis by secreting various growth factors, including glial cell line-derived neurotrophic factor (GDNF), stem cell factor (SCF), and platelet-derived growth factor (PDGF) [6,7,8]. The lactic acid produced by SCs through glycolysis serves as the primary energy source for spermatogenic cells [9,10]. Furthermore, SCs are responsible for phagocytosing dead spermatogenic cells and residual bodies [11,12]. Consequently, SCs have attracted increasing attention in recent research due to their significant role in spermatogenesis.

T-2 toxin is the most toxic member of the type A trichothecenes of mycotoxins, which are produced by the genus Fusarium. This mycotoxin can infiltrate the food chain by contaminating agricultural crops and inducing oxidative stress in various cells, tissues, and organs [13,14]. Exposure to T-2 toxin through oral ingestion, dermal contact, injection, or other routes can result in animal poisoning. Symptoms of T-2 toxin poisoning in animals may include vomiting, diarrhea, anorexia, ataxia, bradycardia, and coagulation disorders [15,16,17]. Furthermore, chronic exposure to T-2 toxin has been linked to multiple severe health disorders in livestock, including immunosuppression, skeletal hypoplasia, nutritional deficiencies, endocrine disruption, and tumorigenesis. These pathological conditions ultimately lead to substantial economic losses in animal production [18,19,20]. Mitochondrial biogenesis is one of the key mechanisms that govern mitochondrial quality and quantity. Disrupted mitochondrial biogenesis can lead to abnormal mitochondrial morphology, elevated oxidative stress levels, reduced cellular metabolism, and ultimately, cell death [21,22]. Mitochondria have been identified as a key organelle targeted by T-2 toxin. For example, in the central nervous system, T-2 toxin exposure can result in perturbations of the mitochondrial respiratory chain complex and mitochondrial biogenesis [23]. Similar effects have also been observed in mouse embryonic stem cells [24]. Recent studies have increasingly demonstrated that T-2 toxin adversely affects the mammalian reproductive system. Exposure to T-2 toxin has been demonstrated to impair oocyte maturation capabilities in mice and pigs by disrupting cytoskeletal dynamics, the endomembrane system, apoptosis/autophagy processes, oxidative stress responses, and epigenetic modifications [25,26,27]. Furthermore, T-2 toxin exposure induces apoptosis and inhibits steroidogenesis in rat and pig ovarian granulosa cells [28,29]. In males, T-2 toxin compromises fertility by disrupting the hypothalamic-pituitary-testis axis and diminishing testicular function in mice [30]. Several investigations have indicated that T-2 toxin can induce apoptosis through oxidative stress in the Sertoli and Leydig cells of mice [31,32].Additionally, T-2 toxin negatively impacts the viability of goat spermatogonial stem cells via autophagy [33]. Moreover, treatment of bovine spermatozoa with T-2 toxin in conjunction with other mycotoxins has been associated with increased oxidative stress and decreased mitochondrial membrane potential [34]. Consequently, it is imperative to investigate the toxic effects of T-2 toxin on the reproductive systems of different species and explore potential preventive measures.

Proanthocyanidins (PCs) are a broad category of polyphenolic compounds prevalent in various plant species. These compounds are recognized for their significant antioxidant properties, which enable effective free radical neutralization [35,36]. Mitochondria are considered important targets of proanthocyanidins (PCs). For instance, grape seed proanthocyanidin extract (GSPs) has been shown to regulate mitochondrial biogenesis and dynamics in diabetic kidney disease [37]. Additionally, in the context of type 2 diabetes mellitus, PCs can significantly improve mitochondrial function [38]. Numerous studies have demonstrated that PCs can mitigate oxidative stress-induced damage to mammalian reproductive systems. Research findings indicate that PCs confer protective effects against zearalenone-induced oxidative damage in the testes and SCs of mice and pigs [39,40]. Moreover, GSP has been demonstrated to alleviate arsenic-induced oxidative reproductive toxicity in male mice [41]. Nevertheless, the potential mitigating effects of PCs on T-2 toxin-induced cytotoxicity and the underlying mechanisms remain elusive. The yak (*Bos grunniens*) is a unique livestock species that inhabits the Tibetan Plateau and serves as a crucial resource for the herdsmen in this region [42,43]. Under traditional husbandry practices, such as grazing and suboptimal feed storage conditions, yaks may face a higher risk of mycotoxin contamination. Sertoli cells are somatic cells that play an essential role in maintaining spermatogenesis in the testes. The isolation, purification, and culture of Sertoli cells in yaks have been well-established, rendering them an ideal cellular model for investigating reproductive toxicology in this species [44,45]. Nonetheless, the potential toxic effects of T-2 toxin on yak Sertoli cells and the identification of effective countermeasures remain to be systematically elucidated. This study had two primary objectives: first, to investigate the toxic effects of T-2 toxin on yak SCs, and second, to evaluate the potential of PCs to mitigate this cytotoxicity. Furthermore, the underlying molecular mechanisms were explored. The findings are expected to enhance our understanding of the potential role of T-2 toxins in impairing yak reproduction, while also offering novel insights into strategies for preventing their cytotoxic effects and supporting the application of PCs in male reproductive health.

## 2. Materials and Methods

### 2.1. Animals

Healthy Maiwa yaks, aged between 3 and 5 years and of comparable size, were selected as experimental subjects at the Qingbaijiang Slaughterhouse located in Chengdu, Sichuan Province. Testicular tissues were collected immediately post-slaughter. Samples intended for real-time polymerase chain reaction (RT-PCR) analysis were promptly placed in liquid nitrogen at –196 °C, transported to the laboratory, and stored at −80 °C. Testes designated for Sertoli cell isolation were preserved in sterile physiological saline supplemented with 80 IU/mL penicillin and 100 IU/mL streptomycin(Biosharp, BL505A, Hefei, China) and transported to the laboratory within 2 h. All experimental procedures were conducted following the permit guidelines established by Southwest Minzu University, and all animal handling was performed in compliance with the ethical standards set forth by the Animal Care and Ethics Committee of Southwest Minzu University (approval code: SMU202501020).

### 2.2. Sertoli Cell Isolation, Culture, and Treatment

Testes were washed three times with pre-warmed sterile 1× PBS (diluted from 10× PBS, Biosharp, BL316A, Hefei, Anhui, China) containing 1% penicillin–streptomycin, immersed in 75% ethanol for 30 s for surface disinfection, and subsequently transferred to a sterile workspace. After carefully removing the tunica vaginalis and tunica albuginea, the testes were washed three additional times with PBS. Approximately 2 cm^3^ of testicular parenchyma was excised, minced into a chyme-like consistency (approximately 8 mL per tube) using ophthalmic scissors, and transferred to a 50 mL centrifuge tube. Five volumes of digestion solution consisting of DMEM/F12 (Gibco, C11330500BT, Grand Island, NY, USA) containing 1 mg/mL collagenase type IV (BioFroxx, 2091GR001, Einhausen, Germany) and 50 µg/mL DNase I (Beyotime, D7073, Shanghai, China) were added, following which the mixture was incubated at 37 °C in a water bath for 40 min, with vigorous shaking every 3 min. Following digestion, the supernatant was carefully removed. The remaining tissue was resuspended in 25 mL of 0.25% trypsin-EDTA (Gibco, 25200072, Grand Island, NY, USA) and incubated at 37 °C in an incubator for 10 min with intermittent shaking every 3 min. Digestion was terminated by the addition of 25 mL of culture medium (DMEM/F12 supplemented with 5% horse serum (Gibco, 16050122, Grand Island, NY, USA), 2.5% fetal bovine serum (Gibco, 10099-141, Grand Island, NY, USA), and 1% penicillin–streptomycin). Next, the suspension was sequentially filtered through 100-mesh and 200-mesh nylon sieves. The filtrate was collected, transferred to centrifuge tubes, and centrifuged at 3200× *g* for 5 min. Afterward, the supernatant was discarded, and the cell pellet was resuspended in culture medium and seeded into T25 culture flasks. Cells were cultured at 37 °C in a humidified atmosphere containing 5% CO_2_. To purify Sertoli cells, the medium was replaced 8 h after seeding using a differential adherence technique, followed by medium changes every 24 h thereafter. Cells were passaged, and the second-passage cells were utilized for subsequent experiments.

Proanthocyanidins (PCs) (Solarbio, P7230, Beijing, China) were initially dissolved in DMSO (Solarbio, D8371, Beijing, China) prior to treatment, whilst T-2 toxin was dissolved directly in culture medium. Passaged yak SCs were seeded into appropriate culture plates and maintained at 37 °C in a 5% CO_2_ incubator. Upon reaching 60–80% confluence, the cells exhibited a relatively dense arrangement, forming one or more layers that were firmly attached to the bottom of the dish and predominantly displaying spindle-shaped and triangular morphologies. Subsequently, the cells were exposed to culture medium containing varying concentrations of T-2 toxin (0, 2, 4, 6, 8, 10, 12, and 14 nM) or PCs. The final concentrations of DMSO in the PC treatment groups were 0, 0.0005%, 0.0025%, 0.005%, 0.01%, 0.02%, 0.05%, and 0.1% (*v*/*v*), corresponding to PC concentrations of 0, 0.5, 2.5, 5, 10, 20, 50, and 100 µg/mL, respectively. All cells were incubated at 37 °C with 5% CO_2_ for 24 h prior to subsequent experiments.

The concentration range for T-2 toxin (0, 2, 4, 6, 8, 10, 12, and 14 nM) was designed to cover a broad spectrum from non-toxic to highly toxic levels, based on preliminary experiments and previous studies involving Sertoli cells and other cell types [32,46]. The concentration range for PCs (0, 0.5, 2.5, 5, 10, 20, 50, and 100 µg/mL) was designed to include both low and high doses based on preliminary experiments and previous reports on testicular cells [40].

### 2.3. Immunofluorescence Staining for Sertoli Cell Identification

To confirm the identity and purity of isolated Sertoli cells, immunofluorescence staining was performed using Sertoli cell-specific markers WT1 and SOX9. Briefly, cells were seeded in 24-well plates and cultured until 60–70% confluence was achieved. After washing with PBS, cells were fixed with 4% paraformaldehyde (Biosharp, BL539A, Beijing, China) for 30 min, permeabilized with 0.2% Triton X-100 (Sigma, T8200, St. Louis, MO, USA) for 20 min, and blocked with 5% bovine serum albumin (BSA) (Sigma, A8020, St. Louis, MO, USA) for 1 h. The cells were then incubated overnight at 4 °C with primary antibodies against WT1 and SOX9, followed by incubation with fluorophore-conjugated secondary antibodies (dilution ratio 1:200) for 1.5 h at room temperature in the dark. Nuclei were counterstained with DAPI (Biosharp, BL105A, Beijing, China) for 10 min. Fluorescence images were captured using an inverted fluorescence microscope (Zeiss, Oberkochen, Germany). Sertoli cell purity was assessed by calculating the proportion of WT1/SOX9-positive cells relative to the total number of DAPI-stained nuclei. Each treatment group consisted of three independent biological replicates, with each biological replicate comprising three technical replicates. Antibodies used in this study are listed in Table S1.

### 2.4. Cell Counting Kit-8 (CCK-8) Assay

The cells were cultured in 96-well plates at a density of approximately 1 × 10^4^ cells/well. Subsequently, the cell proliferation rate was assessed using a CCK-8 Kit (MCE, HY-K0301, Shanghai, China) following the manufacturer’s instructions. The CCK-8 assay, based on the reduction in WST-8 to a formazan dye by viable cells, was used to assess cell proliferation, with absorbance measured at 450 nm (Thermo Scientific™, Waltham, MA, USA). The values were then normalized to the 0 nM T-2 toxin treatment group or control group (set as 100%), and the percentage changes were calculated based on the normalized mean values. Each treatment group consisted of six independent biological replicates, and each biological replicate included three technical replicates.

The half-maximal inhibitory concentration (IC_50_) of T-2 toxin on yak SCs was calculated based on the cell viability data obtained from the CCK-8 assay. Cell survival rates were imported into GraphPad Prism software (version 10.0, GraphPad Software, San Diego, CA, USA), with drug concentrations (X) and corresponding cell survival percentages (Y) as variables. The concentrations were logarithmically transformed [i.e., X = log(X)], and nonlinear regression analysis was performed using the “log(inhibitor) vs. normalized response—Variable slope” model. Each treatment group consisted of six independent biological replicates, and each biological replicate included three technical replicates.

### 2.5. 5-Ethynyl-2′-Deoxyuridine (EdU) Staining

EdU staining of yak SCs subjected to different treatments was performed using the BeyoClick™ EdU Cell Proliferation Kit with Alexa Fluor 488 (Beyotime, C0071S, Shanghai, China), following the manufacturer’s protocol. SCs were incubated with 10 µM EdU solution for 2 h. Subsequently, the cells were fixed in 4% PFA and permeabilized with 0.3% Triton X-100 at room temperature for 15 min. After washing thrice with wash buffer (PBS containing 3% BSA), a click reaction solution was added, and the cells were incubated in the dark for 30 min. The nuclei were stained with Hoechst (Solarbio, C0031, Beijing, China), and images were captured using a ZEISS microscope (Zeiss, LSM800, Oberkochen, Germany). Each treatment group consisted of three independent biological replicates, and each biological replicate included three technical replicates. For each sample, three random fields were photographed. The proliferation rate was calculated as the percentage of EdU-positive cells relative to the total number of cells within each field. The values were then normalized to the control group (set as 100%), and the percentage changes were calculated based on the normalized mean values.

### 2.6. Terminal Deoxynucleotidyl Transferase (TUNEL) Assay

The cells were cultured in 24-well plates. Subsequently, the cells were exposed to different drugs for 24 h. The cells were then fixed in 1 mL of 4% paraformaldehyde for 20 min and rinsed thrice with PBS. The cells were then permeabilized with 1% Triton X-100 for 5 min. TUNEL assay was performed using a commercially available assay kit (KeyGen Biotech, KGA1406, Nanjing, Jiangsu, China) following the manufacturer’s protocol. Fluorescence was observed using a fluorescence microscope (Zeiss, Oberkochen, Germany). Each treatment group consisted of three independent biological replicates, with each biological replicate comprising three technical replicates. For each sample, images were captured from three randomly selected fields. The apoptosis rate was calculated as the proportion of TUNEL-positive cells relative to the total number of cells within each field. The values were then normalized to the control group (set as 100%), and the percentage changes were calculated based on the normalized mean values.

### 2.7. γ-H2AX Immunofluorescence Staining

DNA damage was assessed using a DNA Damage/γ-H2AX Analysis Kit (KeyGen Biotech, KGA9505-100, Nanjing, Jiangsu, China) according to the manufacturer’s instructions. Yak SCs cultured in 24-well plates were fixed with 4% paraformaldehyde for 15 min, permeabilized with 0.2% Triton X-100 for 15 min, and blocked with 3% BSA for 60 min. They were then incubated with primary antibody against γ-H2AX for 60 min at room temperature, followed by incubation with fluorophore-conjugated secondary antibody and Hoechst 33342 for 60 min in the dark. Images were captured using a fluorescence microscope (Zeiss, Oberkochen, Germany). Each treatment group consisted of three independent biological replicates, with each biological replicate including three technical replicates. For each sample, images were captured from three randomly selected fields. The proportion of γ-H2AX-positive cells was calculated as number of γ-H2AX-positive cells/total number of Hoechst-stained nuclei × 100%. The values were subsequently normalized to the control group (set as 100%), and percentage changes relative to the control or T-2 group were calculated based on the normalized mean values.

### 2.8. Measurement of Reactive Oxygen Species (ROS), Malondialdehyde (MDA), Superoxide Dismutase (SOD), and Catalase (CAT) Activity

Intracellular ROS levels were evaluated using a ROS Assay Kit (Beyotime, s0033s, Shanghai, China). Cells were seeded in 24-well plates, cultured to 60–70% confluence, and treated with drugs for 24 h. After washing with DMEM, cells were incubated with 200 μL of 10 μM DCFH-DA probe at 37 °C for 30 min. Cells were then washed, stained with Hoechst 33258 (Beyotime, C1011, Shanghai, China) for 10 min, and washed again. Fluorescence images were captured using a fluorescence microscope (Zeiss, Oberkochen, Germany). Three biological replicates were included for each treatment group. For each sample, images were acquired from three randomly selected fields. Fluorescence intensity was quantified using ImageJ software (version 1.54p, National Institutes of Health, Bethesda, MD, USA) by measuring the mean fluorescence intensity within the threshold-selected regions. The values were then normalized to the control group (set as 100%), and percentage changes were calculated based on the normalized mean values

For MDA detection, cells were cultured in six-well plates at a density of 5 × 10^5^ cells/mL. After treatment with T-2 toxin and PCs, the original medium was aspirated, and the cells were detached by scraping. The cell suspension was centrifuged at 3200× *g* for 10 min. The supernatant was subsequently discarded, and a small volume of PBS was added to the cell pellet. The cells were disrupted using an ultrasonic cell crusher (JY92-IIN, SCIENTZ, Ningbo, China) in an ice-water bath. The MDA content was quantified using an MDA assay kit (A003, Nanjing Jiancheng Bioengineering Institute, Nanjing, China). SOD and CAT activities were evaluated using a SOD assay kit (Nanjing Jiancheng Bioengineering Institute, A001-2, Nanjing, China) and a CAT activity assay kit (Solarbio, BC0205, Beijing, China), respectively, following the manufacturer’s protocols. Each treatment group consisted of three independent biological replicates, and each biological replicate included three technical replicates.

### 2.9. RNA Extraction, cDNA Synthesis, and Real-Time PCR

Total RNA was extracted from yak SCs (5 × 10^5^–1 × 10^6^ cells per sample) using TRIzol reagent (15596026, Invitrogen, Carlsbad, CA, USA). RNA purity was assessed using a UV spectrophotometer (Shimadzu, BioSpec-nano, Kyoto, Japan). cDNA was synthesized using the PrimeScript™ RT Reagent Kit (RR037A, TaKaRa, Shiga, Japan) according to the manufacturer’s instructions. Real-time PCR was performed in a 20 μL reaction volume containing 10 μL of 2× SYBR Master Mix (TaKaRa, RR420A, Shiga, Japan), 0.5 μL each of forward and reverse primers (10 μM), 1 μL of cDNA template, and ddH_2_O to achieve the final volume. The thermal cycling conditions were as follows: initial denaturation at 95 °C for 3 min, followed by 35 cycles of denaturation at 95 °C for 15 s and annealing/extension at 60 °C for 30 s. GAPDH served as the internal reference gene, and relative gene expression levels were calculated using the 2^−ΔΔCt^ method. Each treatment group consisted of three independent biological replicates, with each biological replicate consisting of three technical replicates. The primer sequences used in this study are listed in Table S2.

### 2.10. Western Blotting

SCs were seeded into 6-well plates at a density of approximately 2 × 10^6^ cells per well. After discarding the culture medium and washing cells with PBS, lysis buffer (100 mM Tris/HCl, pH 7.4, 3% SDS, 10 mM DTT, 17.3% glycerol, and 0.15% bromophenol blue) supplemented with cOmplete™ Protease Inhibitor Cocktail (04693116001, Roche, Basel, Switzerland) was added, and cells were lysed on ice for 30 min. Next, the lysate was centrifuged at 10,010× *g* for 15 min at 4 °C, and the supernatant was collected for further analysis. Protein concentrations were determined using the BCA method with a BCA Protein Assay Kit (Solarbio, PC0020, Beijing, China). The BCA working solution was prepared by mixing BCA reagent and Cu reagent at a ratio of 50:1. To generate the standard curve, 10 μL of BSA standard (5 mg/mL) was diluted with PBS to a volume of 100 μL (final concentration 0.5 mg/mL). The diluted standards were added to a 96-well plate at volumes of 0, 2, 4, 6, 8, 12, 16, and 20 μL per well, and each well was supplemented with PBS to achieve a final volume of 20 μL, corresponding to standard concentrations of 0, 0.05, 0.1, 0.15, 0.2, 0.3, 0.4, and 0.5 mg/mL, respectively. Appropriately diluted samples (20 μL) were introduced into the sample wells. Subsequently, 200 μL of BCA working solution was added to each well, and the plate was incubated at 37 °C for 30 min. Absorbance was measured at 562 nm using a microplate reader (Thermo Fisher Scientific, Multiskan FC, 51119000, Waltham, MA, USA), and protein concentrations were calculated based on the standard curve. Western blot analysis was performed as previously described [32]. Before incubating with the primary antibody, the membrane was cut based on the estimated positions of the target proteins as determined by reference to the molecular weight marker. GAPDH served as an internal control. The gray values of the bands were quantified using TANON GIS software (version 4.2, Tanon Science & Technology Co., Ltd., Shanghai, China). Each treatment group consisted of two independent biological replicates, with each biological replicate comprising three technical replicates. Detailed information regarding antibodies and dilutions used in this study is presented in Table S1.

Due to institutional biosafety protocols requiring the disposal of biohazardous waste, the original membranes for some experiments were discarded after the initial figure preparation. However, the authors confirm that all data presented are genuine and have not been manipulated. High-resolution photographs of the cropped membranes are provided in the Supplementary Materials.

### 2.11. Mitochondrial Membrane Potential (MMP) Detection

Cultured cells were seeded into 24-well plates and treated with a Mitochondrial Membrane Potential Assay Kit (Beyotime, C2006, Shanghai, China) according to the manufacturer’s instructions. Cells were incubated with the fluorescent probe JC-1 (5,5′,6,6′-tetrachloro-1,1′,3,3′-tetraethylbenzimidazolylcarbocyanine) at 37 °C for 20 min. Following incubation, the supernatant was removed, and the cells were washed twice with diluted JC-1 staining buffer (1×). Fluorescence signals were observed using a fluorescence microscope (Zeiss, Oberkochen, Germany). JC-1 aggregates (red fluorescence) were detected at excitation/emission wavelengths of 520/595 nm, while JC-1 monomers (green fluorescence) were detected at 490/530 nm. Green fluorescence reflects a decrease in mitochondrial membrane potential, suggestive of early-stage apoptosis. In contrast, red fluorescence indicates intact mitochondrial membrane potential and healthy cellular status. Fluorescence intensity was quantified using ImageJ software (version 1.54p, NIH, Bethesda, MD, USA) by measuring the mean fluorescence intensity within threshold-selected regions. The ratio of red to green fluorescence intensity was calculated, and the relative ratio was normalized to the control group (set as 1). Each treatment group consisted of three independent biological replicates, with each biological replicate consisting of three technical replicates.

### 2.12. Transmission Electron Microscopy (TEM)

The treated cells were collected and centrifuged at 3200× *g* for 5 min. Following centrifugation, the cells were precipitated and subsequently fixed with 2.5% glutaraldehyde and 1% osmium tetroxide for 2 h. The samples were then dehydrated using acetone at varying concentrations in a stepwise manner. Subsequently, acetone was mixed with the embedding resin Epon-812 (02660, SPI, Wilmington, DE, USA) at ratios of 3:1, 1:1, and 1:3 to prepare different concentrations of infiltrating agents. The samples were infiltrated progressively and embedded in Epon-812. Ultrathin sections (60–90 nm) were prepared using an ultramicrotome (UC7rt, LEICA, Wetzlar, Germany) and mounted onto copper grids. The samples were stained with uranyl acetate for 10–15 min, followed by staining with lead citrate for 1–2 min, and finally photographed using a transmission electron microscope (JEM-1400FLASH, JEOL, Tokyo, Japan). Each treatment group consisted of three independent biological replicates, and each biological replicate included three technical replicates.

Mitochondria were considered abnormal if they exhibited one or more of the following features: swelling (increased volume with reduced matrix electron density), vacuolation (presence of empty areas within the matrix), cristae disruption (linearization of cristae, abnormal angular configurations, concentric layering of cristae membranes, or loss of cristae), membrane damage (discontinuous outer membrane or detachment of membranes), abnormal inclusions (paracrystalline inclusions in the matrix), or abnormal morphology (donut-shaped mitochondria) [47,48]. The percentage of abnormal mitochondria was calculated as (number of abnormal mitochondria/total number of mitochondria counted) × 100%. The values were then normalized to the control group (set as 100%), and the percentage changes were calculated based on the normalized mean values.

### 2.13. Measurement of Intracellular ATP Levels

Yak SCs were seeded in 6-well plates at a density of 5 × 10^5^ cells/mL (2 mL per well). After 24 h of treatment, the culture medium was removed, and cells were harvested using a cell scraper. The cell suspension was collected and centrifuged at 3200× *g* for 10 min at room temperature. Then, the supernatant was discarded, and the cell pellet was resuspended in 300 μL of pre-cooled double-distilled water, followed by ultrasonication in an ice-water bath. The lysate was heated in a boiling water bath for 10 min and then vortexed for 1 min. After centrifugation, the supernatant was collected for ATP quantification using an ATP assay kit (Beyotime, S0026, Shanghai, China) according to the manufacturer’s instructions. ATP levels were normalized to the mean value of the control group, which was set as 1. Each treatment group consisted of three independent biological replicates, with each biological replicate comprising three technical replicates.

### 2.14. Statistical Analysis

Statistical analyses were performed using SPSS software (version 26.0, IBM Corp., Armonk, NY, USA), and graphical representations were generated using Prism software (version 10.0, GraphPad Software, San Diego, CA, USA). Statistical comparisons were performed using Student’s unpaired *t*-test or one-way ANOVA (with Tukey’s multiple comparisons test as the post hoc test). All data are expressed as mean ± standard error (mean ± SEM). Statistical significance was set at * *p* < 0.05, ** *p* < 0.01, and *** *p* < 0.001.

## 3. Results

### 3.1. T-2 Toxin Impaired Cell Survival and Elevated ROS Levels in Yak SCs

**Figure 1:**
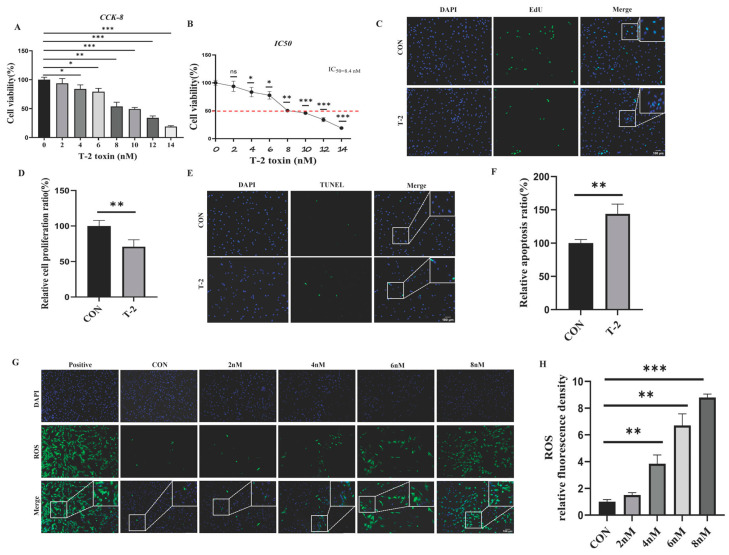
Cytotoxicity detection of T-2 toxin on yak SC viability. (**A**) CCK-8 assay was performed to detect the proliferation rates of yak SCs treated with different concentrations (0, 2, 4, 6, 8, 10, 12, and 14 nM) of T-2 toxin after 24 h. (*n* = 6) (**B**) Cell survival curves of yak SCs treated with different concentrations of T-2 toxin (*n* = 6). (**C**) Cell proliferation was detected by EdU staining in the control (CON) and T-2 groups (*n* = 6). (**D**) Optical density analysis in (**C**) (*n* = 3). (**E**) Apoptotic cells were detected by TUNEL staining in the control and 8 nM T-2 toxin-treated groups (*n* = 3). (**F**) Optical density analysis revealed in (**E**) (*n* = 3). (**G**) Detection of ROS levels in yak SCs treated with different concentrations of T-2 toxin by treating cells with DCFH-DA diacetate (*n* = 3). (**H**) ROS levels in (**G**) were quantified by measuring the fluorescence intensity of DCFH-DA (*n* = 3). For (**A**,**B**,**H**), one-way ANOVA was used (with Tukey’s multiple comparison test as the post hoc test). For (**D**,**F**), Student’s unpaired *t*-test was used. All data are expressed as mean ± SEM. * *p* < 0.05, ** *p* < 0.01, and *** *p* < 0.001 indicated statistical significance at different levels.

Yak SCs were successfully isolated and identified by detecting specific Sertoli cell marker proteins, Wilms tumor1 transcription factor (WT1), and SRY-box transcription factor 9 (SOX9) (Figure S1). Subsequently, the viability of yak SCs treated with varying concentrations of T-2 toxin (0, 2, 4, 6, 8, 10, 12, and 14 nM) was assessed using the CCK-8 assay. The results revealed a significant reduction in the viability of SCs as the concentration of T-2 toxin increased, with a calculated half-maximal inhibitory concentration (IC_50_) of 8.4 nM (Figure 1A,B). Additionally, EdU and TUNEL staining provided further evidence that 8 nM T-2 toxin treatment (T-2 group) inhibited cell proliferation and induced apoptosis in yak SCs (Figure 1C–F). Furthermore, ROS levels in yak SCs exposed to different concentrations of T-2 toxin (0, 2, 4, 6, and 8 nM) were measured and quantified. The findings demonstrated a positive correlation between ROS levels and T-2 toxin concentration (Figure 1G,H). Collectively, these results indicate that T-2 toxin adversely affects the viability of yak SCs and induces oxidative stress.

### 3.2. PCs Inhibited Cellular Apoptosis and Mitigated Functional Impairments in Yak SCs Exposed to T-2 Toxin

**Figure 2:**
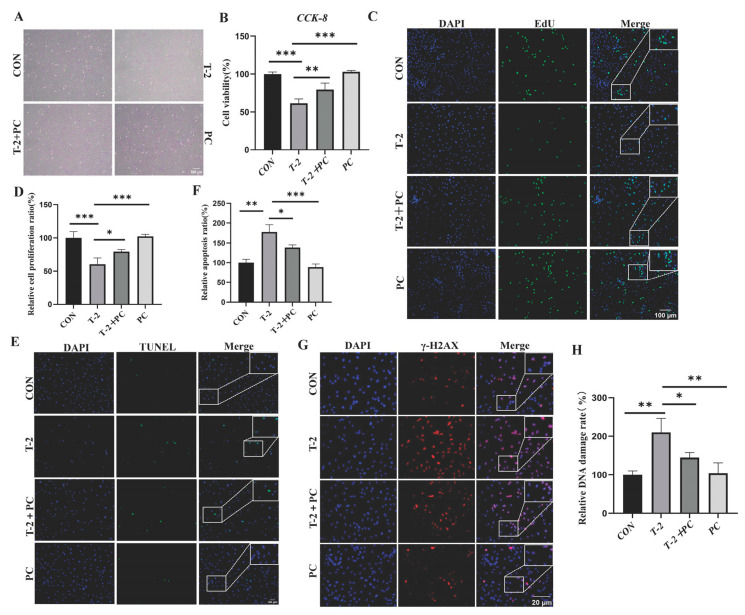
Effect of PCs on yak SC viability treated with T-2 toxin. (**A**) Representative images of cell morphology in the CON, T-2, T-2 + PC, and PC groups. (**B**) CCK-8 assay was performed to assess the cell proliferation rates across groups (*n* = 6). (**C**) Cell proliferation was detected via EdU staining in the different groups (*n* = 3). (**D**) Quantitative analysis of optical density in (**C**) (*n* = 3). (**E**) Apoptotic cells were detected using TUNEL staining across different groups (*n* = 3). (**F**) Quantitative analysis of optical density in (**E**) (*n* = 3). (**G**) γ-H2AX staining was performed to examine DNA damage in the different groups (*n* = 3). (**H**) Proportion of γ-H2AX positive-stained cells in (**G**) (*n* = 3). Data are expressed as mean ± SEM. * *p* < 0.05, ** *p* < 0.01, and *** *p* < 0.001 indicated statistical significance at different levels. One-way ANOVA was used (with Tukey’s multiple comparison test as the post hoc test).

To investigate the effects of PCs on yak SCs treated with T-2 toxin, cell viability was evaluated following exposure to various concentrations of PCs (0, 0.5, 2.5, 5, 10, 20, 50, and 100 µg/mL). Initially, cell viability increased with higher concentrations of PCs, peaking at 10 µg/mL before declining (Figure S2). Further analysis indicated that co-treatment with 8 nM T-2 toxin and 10 µg/mL PCs (T-2 + PC group), together with the 10 µg/mL PC treatment group (PC group), significantly enhanced cell viability compared to the T-2 group alone (Figure 2A,B). Additionally, EdU staining revealed a notable improvement in cell proliferation within the T-2 + PC group relative to the T-2 group alone (Figure 2C,D). Cell apoptosis was assessed using the TUNEL assay, which demonstrated that treatment with PCs significantly mitigated T-2 toxin-induced apoptosis in yak SCs (Figure 2E,F). Considering that DNA damage is a primary instigator of cell apoptosis, the extent of DNA damage was evaluated through immunostaining for phosphorylated histone H2AX (γ-H2AX), a well-established surrogate marker for DNA damage. The proportion of SCs exhibiting DNA damage was significantly elevated in the T-2 group by approximately 109.9% compared to the control group. Contrarily, treatment with PCs resulted in a significant reduction in DNA damage levels by 31.16% in T-2 toxin-treated cells when compared to the T-2 group (Figure 2G,H).

**Figure 3:**
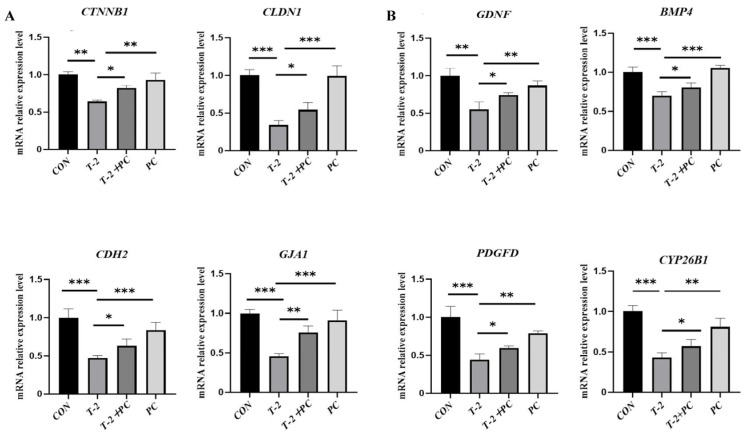
Impact of PC treatment on the expression of Sertoli cell function-related genes in T-2 toxin-treated yak SCs. (**A**) Relative mRNA levels of key Sertoli cell-secreted factors responsible for spermatogenesis in the CON, T-2, T-2 + PC, and PC groups (*n* = 3). (**B**) Relative mRNA levels of genes encoding BTB-related proteins across different groups (*n* = 3). Data are expressed as mean ± SEM. * *p* < 0.05, ** *p* < 0.01, and *** *p* < 0.001 indicated statistical significance at different levels; one-way ANOVA was used (with Tukey’s multiple comparison test as the post hoc test).

SCs are vital for supporting spermatogenesis by forming the BTB and facilitating paracrine signaling. This study evaluated the mRNA expression levels of various BTB components, including N-cadherin, catenin beta 1 (*CTNNB1*), claudin 1 (*CLDN1*), cadherin 2 (*CDH2*), and gap junction protein alpha 1 (*GJA1*). Additionally, several spermatogenesis-related factors secreted by SCs, including glial cell line-derived neurotrophic factor (*GDNF*), bone morphogenetic protein 4 (*BMP4*), platelet-derived growth factor D (*PDGFD*), and cytochrome P450, family 26, subfamily b, polypeptide 1 (*CYP26B1*), were assessed. The results indicated a significant suppression of gene expression in the T-2 group; however, co-treatment with T-2 and PCs was associated with a partial normalization of these expression levels (Figure 3). These findings collectively indicate that PCs correlate with modified expression profiles of genes related to Sertoli cell function.

### 3.3. PCs Mitigated Excessive Oxidative Stress Induced by T-2 Toxin in Yak SCs, an Effect Correlated with Upregulation of the NFE2-like bZIP Transcription Factor 2 (NRF2)/Heme Oxygenase-1 (HO-1) Signaling Pathway

**Figure 4:**
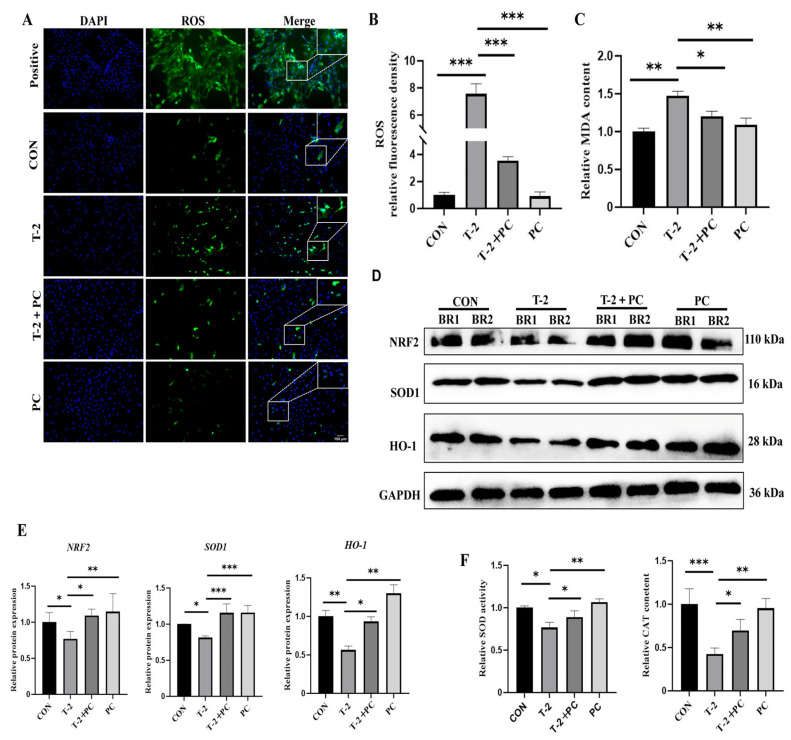
PC treatment attenuates T-2-induced oxidative stress and improves antioxidant ability in yak SCs. (**A**) Detection of ROS levels in CON, T-2, T-2 + PC, and PC groups by treating cells with DCFH-DA diacetate, *n* = 3. (**B**) Quantification of ROS levels in (**A**) by measuring the fluorescence intensity of DCFH-DA (*n* = 3). (**C**) MDA levels in the CON, T-2, T-2 + PC, and PC groups (*n* = 3). (**D**) Protein expression levels of the antioxidant proteins NRF2, SOD1, and HO-1 across groups. BR1 and BR2 represent two independent biological replicates (*n* = 2). (**E**) Quantitative analysis of optical density in (**D**) (*n* = 3). (**F**) Activities of the antioxidant enzymes SOD and CAT across groups (*n* = 3). For ROS, MDA, SOD, and CAT data, values are expressed as mean ± SEM; statistical significance was determined by one-way ANOVA followed by Tukey’s test. For Western blot data for NRF2, SOD1, and HO-1, values are expressed as mean ± SEM; statistical comparisons between two groups were performed using Student’s *t*-test. * *p* < 0.05, ** *p* < 0.01, and *** *p* < 0.001 indicated statistical significance at different levels.

Given that T-2 toxin induces excessive oxidative stress in yak SCs, and PCs serve as potent antioxidants, ROS levels in yak SCs following treatment with a combination of PCs and T-2 toxin were assessed. Compared to the T-2 group, ROS levels were reduced by 53.1% in the T-2 + PC group (Figure 4A,B). Moreover, MDA, a biomarker of lipid peroxidation and oxidative stress, was significantly increased in the T-2 group compared to that in the control group. In contrast, MDA levels decreased significantly in the T-2 + PC group (Figure 4C). The *NRF2*/HO-1 signaling pathway is vital for alleviating oxidative stress. The protein expression of several components within this pathway, including NRF2, HO-1, and SOD1, was assessed. The results indicated that treatment with T-2 toxin significantly decreased the expression of NRF2, HO-1, and SOD1. However, co-treatment with PCs was associated with a reversal of these expression changes in yak SCs (Figure 4D,E and Figure S3). Furthermore, the activities of the antioxidant enzymes SOD and CAT were significantly elevated in the T-2 + PC group compared to the T-2 group (Figure 4F).

### 3.4. PCs Alleviated the Cytotoxic Effects in Yak SCs Exposed to T-2 Toxin by Repressing Mitochondria-Mediated Apoptotic Pathway

**Figure 5:**
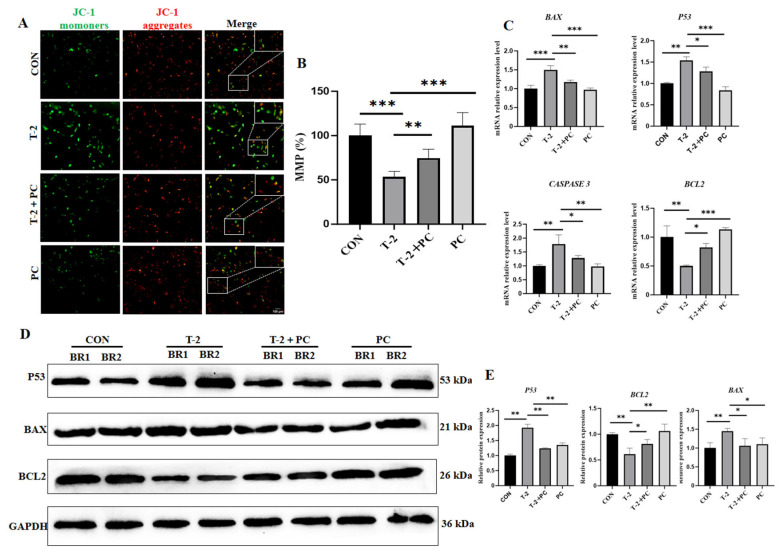
PC treatment alleviates T-2 toxin-induced cytotoxicity in yak SCs by inhibiting the mitochondria-induced apoptotic pathway. (**A**) MMP was detected using the JC-1 fluorescent probe in CON, T-2, T-2 + PC, and PC groups. JC-1-monomers and JC-1-aggregates produce green and red fluorescence, respectively (*n* = 3). (**B**) Quantification of MMP in (**A**) by measuring the ratio of the fluorescence intensity of JC-1-aggregates to JC-1-monomers (*n* = 3). (**C**) Relative mRNA levels of key members of the mitochondria-mediated apoptotic pathway across groups (*n* = 3). (**D**) Protein expression levels of members of the mitochondria-mediated apoptotic pathway, BAX and BCL2, across groups (*n* = 2). (**E**) Quantitative analysis of optical density corresponding to the Western blot results shown in (**D**) (*n* = 2). MMP and qPCR data were analyzed by one-way ANOVA with Tukey’s test. Western blot data for P53, BAX, and BCL2 were analyzed by Student’s *t*-test. Data are shown as mean ± SEM. * *p* < 0.05, ** *p* < 0.01, and *** *p* < 0.001.

Given that mitochondria are the primary targets of T-2 toxin and that mitochondrial dysfunction is a secondary effect responsible for T-2 toxin-induced ROS generation, the mitochondrial membrane potential (MMP) in yak SCs subjected to different treatments was investigated. The results indicated that T-2 toxin treatment resulted in a significant decrease in MMP of 46.6% compared to the control group. Contrarily, the T-2 + PC group exhibited a 40.0% increase in MMP relative to the T-2 group, suggesting that PCs may mitigate the damage to the MMP caused by the T-2 toxin (Figure 5A,B). Furthermore, the expression levels of genes associated with the mitochondria-mediated apoptotic pathway, including *P53*, B-cell lymphoma-2 (*BCL2*)*, BCL2*-associated X, apoptosis regulator (*BAX*), and cysteine-dependent aspartate-specific protease-3 (*CASPASE 3*), were detected. The expressions of the pro-apoptotic genes *P53*, *BAX*, and *CASPASE 3* were significantly increased by 53.9%, 50.0%, and 78.2%, respectively, in the T-2 group compared to the control group. However, the expression of the anti-apoptotic gene *BCL2* was significantly downregulated following T-2 toxin treatment.

The combined treatment of T-2 toxin and PCs significantly reduced the expression of pro-apoptotic genes *P53*, *BAX*, and *CASPASE 3* by 16.7%, 21.5%, and 28.3%, respectively, compared to treatment with T-2 toxin alone. Conversely, the anti-apoptotic gene *BCL2* was significantly increased by 64.6% in the T-2 + PC group relative to the T-2 group (Figure 5C). Besides, the protein expression levels of P53, BCL2, and BAX were assessed, revealing similar results (Figure 5D,E and Figure S3). Collectively, these findings suggest that the protective effect of PCs against T-2 toxin-induced cytotoxicity in yak SCs correlates with the inhibition of the mitochondria-mediated apoptotic pathway.

### 3.5. PCs Mitigated Mitochondrial Dysfunction and Restored the Expression of Key Genes Related to Mitochondrial Biogenesis in Yak SCs Exposed to T-2 Toxin

**Figure 6:**
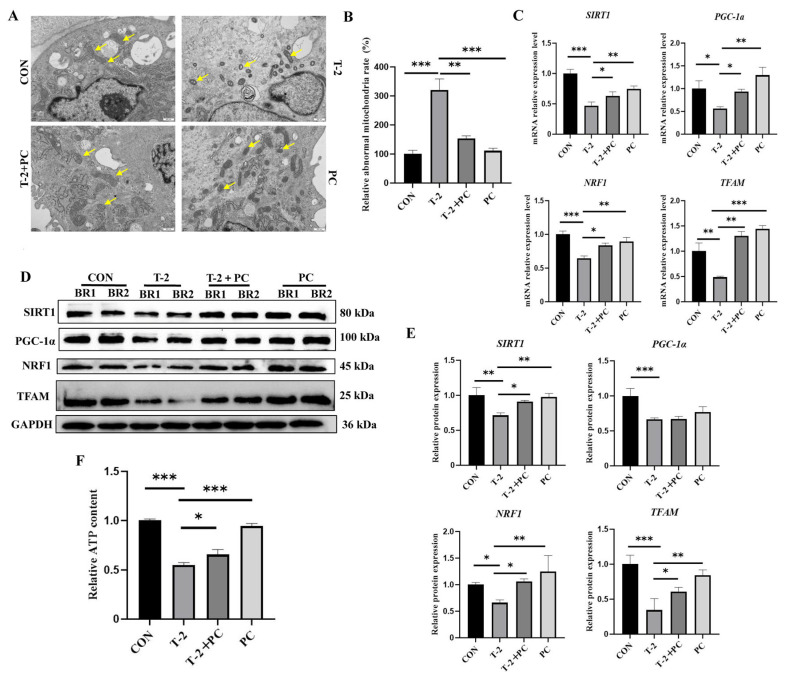
PC treatment improves mitochondrial morphology and biosynthesis in T-2 toxin-treated yak SCs. (**A**) Ultrastructure of the mitochondria in yak SCs of CON, T-2, T-2 + PC, and PC groups as observed by TEM. Mitochondria were detected by yellow arrowheads (*n* = 3). (**B**) Rate of abnormal mitochondria in (**A**) observed by TEM (*n* = 3). (**C**) Relative mRNA levels of several genes related to mitochondrial biosynthesis in CON, T-2, T-2 + PC, and PC groups (*n* = 3). (**D**) Relative protein levels in the different groups (*n* = 2). (**E**) Optical density analysis of the Western blot results shown in (**D**) (*n* = 2). (**F**) Intracellular ATP levels in different groups (*n* = 3). TEM, qPCR, and ATP data were analyzed by one-way ANOVA with Tukey’s test. Western blot data for SIRT1, PGC-1a, NRF1, and TFAM were analyzed by Student’s *t*-test. Data are shown as mean ± SEM. * *p* < 0.05, ** *p* < 0.01, and *** *p* < 0.001.

The morphologies of the mitochondria in yak SCs subjected to different treatments were further investigated. In the control group, the mitochondria were mostly round, oval, or rhabdoid, characterized by intact inner cristae, uniform basal density, and an intact inner membrane. Conversely, the mitochondria in the T-2 group were primarily contracted and vacuolated, displaying shortened and disordered cristae and decreased matrix electron density. Notably, the number of abnormal mitochondria was significantly reduced in the T-2 + PC and PC groups (Figure 6A,B). Furthermore, the expression levels of several key members associated with mitochondrial biogenesis, including sirtuin 1 (*SIRT1*), peroxisome proliferator-activated receptor gamma coactivator 1 alpha (*PGC-1α*), nuclear respiratory factor 1 (*NRF1*), and transcription factor A, mitochondrial *(TFAM*), were assessed. A decrease in the mRNA expression of these components was observed in the T-2 group compared to the control group. However, the combined treatment of T-2 toxin and PCs significantly restored these gene expressions (Figure 6C). Similar results were observed at the protein level (Figure 6D,E and Figure S3).

Additionally, given that mitochondria are the primary organelles responsible for adenosine triphosphate (ATP) production, ATP levels across the different groups were measured. The results indicated that the combined treatment with T-2 toxin and PCs significantly ameliorated the impaired ATP production in yak SCs exposed to T-2 toxin (Figure 6F). Taken together, these findings suggest that PCs ameliorated T-2 toxin-induced defects in mitochondrial structure and function, as well as alterations in the expression of key genes involved in mitochondrial biogenesis in yak SCs.

## 4. Discussion

T-2 toxin, a prevalent environmental contaminant, is the most toxic variant of type A trichothecene mycotoxins [13,49,50]. A significant adverse effect associated with T-2 toxin exposure is reproductive disruption. Evidence of the reproductive toxicity of T-2 toxin includes decreased fertility, alterations in the structure and function of reproductive organs, and impaired gametogenesis in males and females. Additionally, the T-2 toxin interferes with the reproductive endocrine axis and inhibits the synthesis of reproductive hormones [51,52,53]. Despite these concerns, knowledge regarding strategies to mitigate reproductive toxicity is limited. Importantly, to the best of our knowledge, this is the first study to provide evidence that PCs, a diverse group of polyphenolic compounds, can alleviate T-2 toxin-induced toxicity in yak SCs by attenuating oxidative stress and up-regulating the expression of genes related to mitochondrial function and biosynthesis.

Several studies have indicated that the T-2 toxin represses protein expression related to BTB. For example, T-2 toxin in a non-tumorigenic human intestinal cell model resulted in decreased expression of the BTB-related protein claudin-1 [54]. Moreover, the T-2 toxin disrupted the expression of tight junction barrier-related proteins, including occludin, ZO-1, N-cadherin, and β-catenin, in SerW3 cells [55], and exhibited similar effects in the TM4 cell line [32]. In this study, T-2 toxin suppressed the expression of BTB-related genes, specifically *CTNNB1*, *CLDN1*, *CDH2*, and *GJA1*; however, their expression levels were restored following treatment with PCs in yak SCs. Among these, *CTNNB1*, *CLDN1*, and *CDH2* are components of tight junctions, while *GJA1* is integral to gap junction formation. Tight junctions are crucial for establishing barriers and relatively enclosed spaces, whereas gap junctions facilitate the exchange of materials and information between cells [56]. Overall, these findings suggest that T-2 toxin exposure is associated with structural and functional impairments of the BTB in yak testis, which correlate with the downregulation of tight and gap junction protein mRNA levels; conversely, PCs appear to partially alleviate these transcriptional alterations. Nonetheless, further in vivo studies are warranted to validate this hypothesis.

T-2 toxins have been demonstrated to induce cellular and organ damage through ROS generation in various systems, including the circulatory, nervous, and reproductive systems [23,57,58]. The NRF2-HO-1 signaling pathway is crucial for enabling cells to combat oxidative stress and enhancing their intrinsic antioxidant capacity [59,60]. Previous studies have indicated that T-2 toxins can inflict oxidative damage to cells within the nervous system by inhibiting this pathway. Conversely, activation of the NRF2-HO-1 signaling pathway mitigates the oxidative stress toxicity associated with T-2 toxins [23,61]. Furthermore, PCs have been reported to augment cellular antioxidant activity in several cell types by activating the NRF2-HO-1 signaling pathway [62,63,64,65]. This study revealed that compared to the administration of T-2 toxin alone, co-treatment with T-2 toxin and PCs in yak SCs significantly increased the expression levels of multiple components of the NRF2-HO-1 signaling pathway at both the mRNA and protein levels. These results suggest that the protective effect of PCs against T-2 toxin-induced oxidative stress correlates with the upregulation of the NRF2-HO-1 signaling pathway.

Mitochondria are essential organelles that play a critical role in energy production, metabolic processes, and apoptosis regulation. Damaged mitochondria serve as the primary source of ROS within cells and can initiate apoptosis through the mitochondrial pathway. Consequently, the structural and functional integrity of mitochondria, along with appropriate thresholds for mitochondrial biogenesis, is vital for cellular viability [66,67,68]. A previous study demonstrated that T-2 toxin significantly elevated ROS levels in rat pituitary cells. Additionally, this study found that the T-2 toxin inhibited *BCL2* expression while simultaneously increasing the expression of Bax, p53, caspase-3, and caspase-8 [69]. This finding is consistent with our results, which indicate that the reduction in yak SC viability induced by T-2 toxin correlates with alterations in the mitochondria-mediated apoptotic pathway. The *SIRT1*/*PGC1α* signaling pathway is integral to mitochondrial biogenesis and its function [70,71,72]. An earlier study has demonstrated that T-2 toxin at concentrations of 5 and 10 ng/mL down-regulates the expression of key members of the *SIRT1*/*PGC1α* pathway, including *PGC-1α*, *NRF1*, and *TFAM*, in SH-SY5Y cells, ultimately leading to impaired mitochondrial biogenesis [73]. Interestingly, another study revealed that T-2 toxin in the concentration range of 2 nM to 16 nM significantly elevated mitochondrial biogenesis and ROS levels in HepG2 and HEK293T cells, accompanied by a marked increase in *SIRT1* and *PGC1-α* expression levels [46]. The present study unveiled that the 8 nM T-2 toxin exposure in yak SCs is associated with both the enhancement of the mitochondria-mediated apoptotic pathway and impairments in mitochondrial morphology, function, and the expression of genes associated with mitochondrial biogenesis. These results collectively suggest that the effects of T-2 toxin on mitochondrial biogenesis are potentially contingent upon both drug concentration and cell types. Furthermore, treatment with PCs alleviated abnormalities in mitochondrial morphology, biogenesis, and function. These findings suggest that the protective effects on mitochondria may represent a significant mechanism by which PCs alleviate the toxic effects of T-2 toxin in yak SCs. Notably, ROS may be both the cause and consequence of mitochondrial abnormalities. Consequently, besides upregulating the NRF2-HO-1 pathway, PCs may reduce T-2 toxin-induced oxidative stress, an effect that coincides with the improvement of mitochondrial function in yak SCs.

It should be noted that, due to limitations in sample collection, the results of this study were obtained based on second-passage primary yak Sertoli cells. Considering the potential changes in cell status during passaging as well as species differences, whether the protective effects of procyanidins against T-2 toxin-induced cytotoxicity can be extended to other passages of yak Sertoli cells or to Sertoli cells from other species remains to be further investigated in subsequent experiments. Additionally, although in vitro cell models offer advantages for toxicological studies, such as rapid analysis, high-throughput capacity, and controlled experimental conditions, in vivo animal models are irreplaceable in certain key aspects. To begin, in vivo models enable a comprehensive and intuitive assessment of the toxic effects of T-2 toxin and the protective effects of PCs. For example, they allow for observation of behavioral abnormalities in animals induced by T-2 toxin, as well as potential side effects of PC intervention on other organs. Secondly, the actual concentrations of T-2 toxin and PCs reaching target organs or cells, such as the testis and Sertoli cells, are closely related to the animal’s absorption, metabolism, and excretion processes, factors that require further in vivo investigation. Thirdly, the involvement of additional systems, such as the endocrine and immune systems, in mediating the effects of T-2 toxin and proanthocyanidins on the reproductive system can be comprehensively analyzed using in vivo models. Given that yaks are non-model animals with unique living and husbandry conditions, conducting preliminary studies in model animals such as mice may represent a feasible alternative. Additionally, RNA interference or overexpression experiments may be needed to target the Nrf2-HO-1 signaling pathway in PC-treated yak Sertoli cells to further elucidate the molecular mechanism by which PCs alleviate T-2 toxin-induced cytotoxicity.

## 5. Conclusions

**Figure 7:**
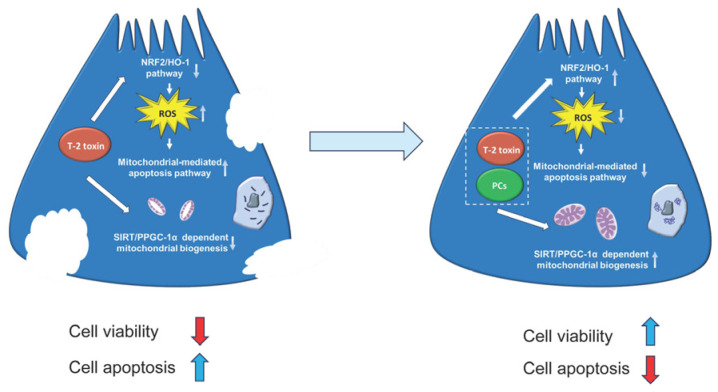
Schematic diagram of the proposed mechanisms by which PCs ameliorate T-2-toxin-induced toxicity in yak SCs.

In summary, this study demonstrated that PCs effectively restored impaired cell viability in yak SCs subjected to T-2 toxin. This protective effect was associated with the attenuation of excessive ROS production, along with improvements in mitochondrial morphology and function, as well as alterations in the expression of genes related to mitochondrial biogenesis (Figure 7). More importantly, these findings provide fundamental data and a framework for further investigations into the reproductive toxicity of T-2 toxin and the mechanisms underlying PC-mediated detoxification in yaks.

## Data Availability

The original contributions presented in this study are included in the article/Supplementary Material. Further inquiries can be directed to the corresponding authors.

## Supplementary Materials

The following supporting information can be downloaded at: https://www.mdpi.com/article/10.3390/antiox15050547/s1, Figure S1: Isolation and identification of yak SCs. Yak SCs were identified by immunostaining with the Sertoli cell marker proteins WT1 and SOX9. Images represent three independent experiments, and three slides were analyzed in each; Figure S2: Effect of PCs on yak SC viability. CCK-8 assay was performed to detect the proliferation rates of yak SCs treated with different PC concentrations; Figure S3: The original Western Blot images. (A–D) Western blot analysis of NRF2 (A), SOD1 (B), HO-1 (C) and GAPDH (D) in yak SCs of CON, T-2, T-2 + PC and PC group corresponding to Figure 4. (E–H) Western blot analysis of P53 (E), BAX (F), BCL2 (G) and GAPDH (H) in yak SCs of CON, T-2, T-2 + PC and PC group to Figure 5. (I-M) Western blot analysis of SIRT1 (I), PGC-1α (J), NRF1 (K), TFAM (L) and GAPDH (M) in yak SCs of CON, T-2, T-2 + PC and PC group to Figure 6. BR1 and BR2 represent two independent biological replications. The size of each membrane was indicated on the images; Table S1: Antibodies; Table S2: Primer sequences.

## Abbreviations

The following abbreviations are used in this manuscript:

PCs	proanthocyanidins
ROS	reactive oxygen species
NRF2	Nuclear factor erythroid derived 2-like
HO-1	heme oxygenase-1
SIRT1	sirtuin 1
PGC-1α	peroxisome proliferator-activated receptor gamma coactivator 1 alpha
SCs	Sertoli cells
BTB	blood-testis barrier
GDNF	glial cell line-derived neurotrophic factor
SCF	stem cell factor
PDGF	platelet-derived growth factor
GSPs	grape seed proanthocyanidins
PCR	real-time polymerase chain reaction
PBS	phosphate-buffered saline
BSA	bovine serum albumin
CCK-8	Cell Counting Kit-8
EdU	5-Ethynyl-2′-deoxyuridine
TUNEL	terminal deoxynucleotidyl transferase
MDA	malondialdehyde
SOD	superoxide dismutase
CAT	catalase
DCFH-DA	2′,7′-dichlorodihydrofluorescein diacetate
GAPDH	glyceraldehyde-3-phosphate dehydrogenase
MMP	mitochondrial membrane potential
JC-1	fluorescence probe 5,5′,6,6′-Tetrachloro-1,1′,3,3′-tetraethyl-imidacarbocyanine
TEM	transmission electron microscopy
WT1	Wilms tumor1 transcription factor
SOX9	SRY-box transcription factor 9
γ-H2AX	phosphorylated histone H2AX
CTNNB1	catenin beta 1
CLDN1	claudin 1
CDH2	cadherin 2
GJA1	gap junction protein alpha 1
BMP4	bone morphogenetic protein 4
PDGFD	platelet-derived growth factor D
CYP26B1	cytochrome P450, family 26, subfamily b, polypeptide 1
BCL2	B-cell lymphoma-2
BAX	BCL2-associated X, apoptosis regulator
CASPASE 3	cysteine-dependent aspartate-specific protease-3
NRF1	nuclear respiratory factor 1
TFAM	transcription factor A, mitochondrial
ATP	adenosine triphosphate
