# Supplementary figures and images for: Proanthocyanidins Alleviate T-2 Toxin-Induced Toxicity in Yak (Bos grunniens) Sertoli Cells by Alleviating Oxidative Stress and Modulating Mitochondrial Biogenesis
Source: Antioxidants (Basel). 2026 Apr 25;15(5):547. doi: 10.3390/antiox15050547 (PMC13203484; doi:10.3390/antiox15050547)

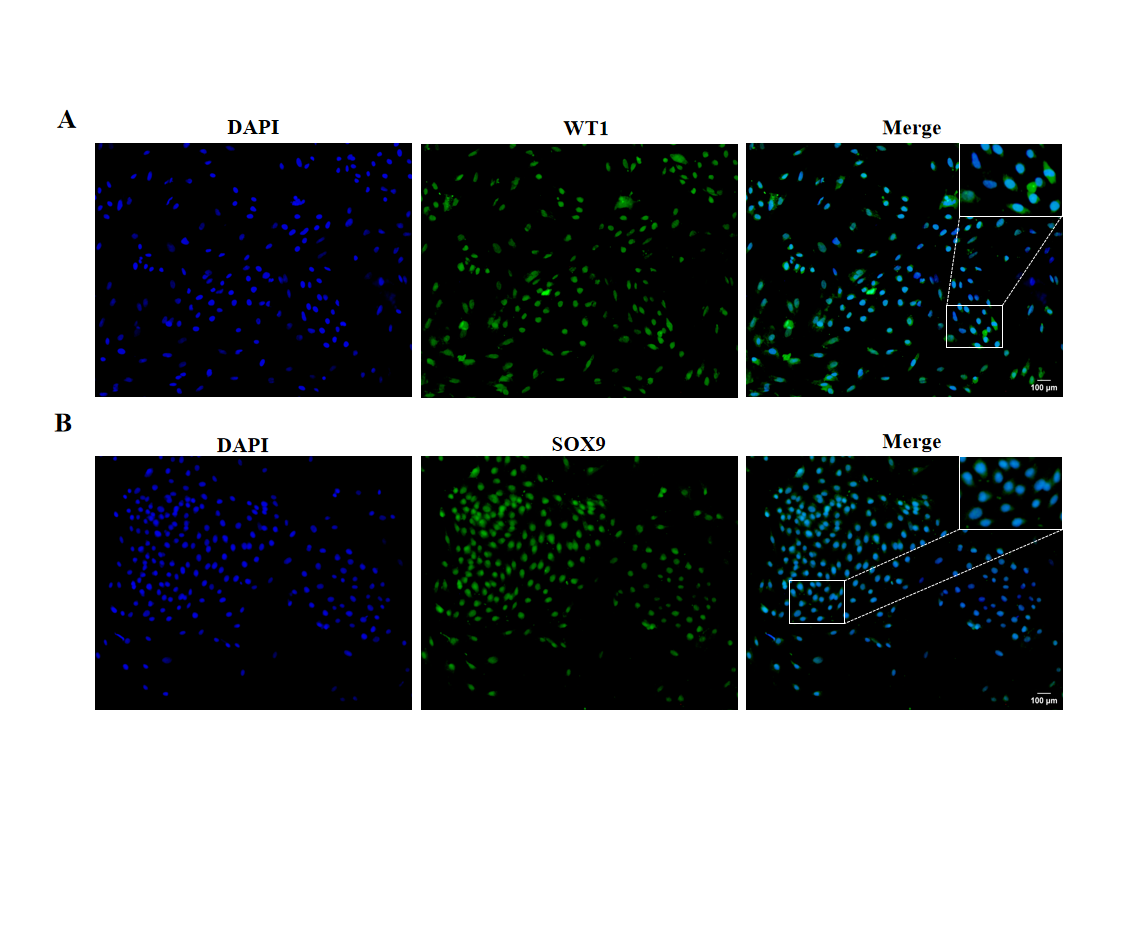

Supplement: Supplementary file 1 [file antioxidants-15-00547-s001.zip › Figure.S1.tif]

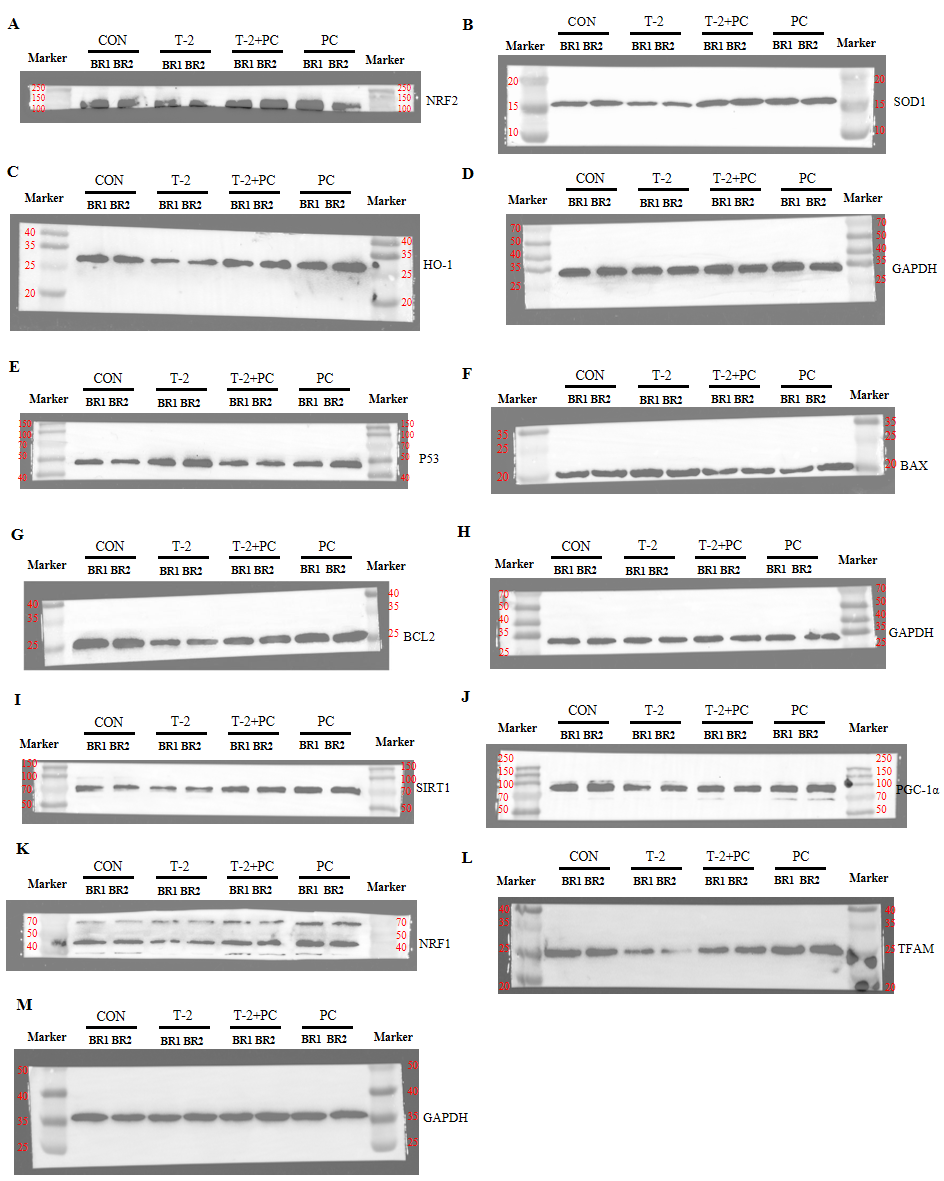

Supplement: Supplementary file 1 [file antioxidants-15-00547-s001.zip › Figure.S3.tif]
